# Liver stiffness assessed by real-time two-dimensional shear wave elastography predicts hypersplenism in patients with Wilson’s disease: a prospective study

**DOI:** 10.1186/s12880-022-00749-x

**Published:** 2022-02-11

**Authors:** Jiajia Wang, Minxia Hu, Qiang Zhu, Lanting Sun

**Affiliations:** 1 Department of Diagnostic Ultrasound, Beijing Tongren Hospital, Capital Medical University, No. 1, Dong Jiao Min Xiang Street, Dongcheng District, Beijing, 100730 China; 2 Department of Ultrasound, The First Affiliated Hospital of Anhui University of Chinese Medicine, Hefei, China; 3 Department of Encephalopathy, The First Affiliated Hospital of Anhui University of Chinese Medicine, Hefei, China

**Keywords:** Wilson’s disease, Hypersplenism, Liver stiffness, Shear wave elastography

## Abstract

**Background:**

The current study aimed to explore the value of liver stiffness assessed by two-dimensional real-time shear wave elastography (2D-SWE) to predict hypersplenism occurrence in Wilson’s disease (WD) patients.

**Methods:**

Ninety WD patients were enrolled in this prospective study between May 2018 and December 2018. Baseline clinical data and ultrasound imaging including 2D-SWE liver stiffness of WD patients were collected. After enrollment, patients had follow-ups for 24 months or until they developed hypersplenism. The hypersplenism risk factors were determined using Cox regressions and receiver operating characteristic curves (ROC).

**Results:**

Twenty-nine (32.2%) patients developed hypersplenism. Age, portal vein diameter, and liver stiffness were independent hypersplenism risk factors in WD patients. The cutoff value of liver stiffness to predict hypersplenism was 10.45 kPa, with sensitivity and specificity of 75.9% and 73.8%, respectively. Patients were divided into two groups according to liver stiffness: ≥ 10.45 kPa (57.9% with hypersplenism) or < 10.45 kPa (13.5% with hypersplenism). The median time between enrollment and hypersplenism development was 15 months vs. 22 months (*p* < 0.001) for the two groups, respectively.

**Conclusion:**

The measurement of liver stiffness by 2D-SWE can be a reliable hypersplenism predictor in WD patients. Therefore, dynamic monitoring of WD patients using 2D-SWE is crucial for the early diagnosis of hypersplenism.

## Background

Wilson’s disease (WD) is a rare autosomal recessive genetic disease [1]. In WD patients, 75% have liver involvement [2], and 34% eventually develop cirrhosis and hypersplenism [3]. WD patients are 4.4 times more likely to develop hypersplenism compared to hepatitis B patients [4]. In liver disease patients, hypersplenism development is determined when they present a leukocyte count < 3500/µL and/or a platelet count < 7.5 × 104/µL [5]. After hypersplenism development, WD patients need be treated with splenectomy [6]. However, patients can have complications such as portal vein thrombosis (PVT) [7] and overwhelming post-splenectomy infection (OPSI) after splenectomy. Also, the mortality rate related to OPSI can be as high as 50% [8]. Therefore, it is of clinical importance to develop techniques to predict whether an individual WD patient will develop hypersplenism. Early intervention with splenic artery embolization can be used to decelerate hypersplenism progression [9].

In previous studies, the assessment of splenic blood flow using four-dimensional (4D) flow magnetic resonance imaging (MRI) has been reported to predict hypersplenism [10]. Patients with high compliance are required in the 4D flow MRI evaluation of splenic blood flow. However, some WD patients could not tolerate MRI examination due to neurological and mental symptoms developed during disease progression [1]. It has been reported that the assessment of liver stiffness by real-time two-dimensional shear wave elastography (2D-SWE) can be applied to predict various hepatic adverse events such as liver cancer caused by hepatitis B and liver failure caused by liver cancer [11, 12], as well as to evaluate whether hepatic patients have complicated portal hypertension [13–15]. The Baveno VI criteria (2015) suggests that transient elastography (TE) can clinically identify significant portal hypertension (CSPH) (1b; A) [16]. On this basis, Fofiu R raised 2D-SWE had a good performance to predict high-risk varices (HRV),) including esophageal varices (EV) and gastric varices (GV) in advanced chronic liver disease (cACLD) [17]. However, no attention has yet been paid to the value of 2D-SWE to predict hypersplenism in WD patients mainly due to the rarity of this disease.

Therefore, in the current work, we conducted a prospective longitudinal cohort study to investigate whether liver stiffness assessment by 2D-SWE can be applied to predict hypersplenism in WD patients.

## Methods

### Patients

Consecutive WD patients admitted to the Encephalopathy Center of the First Affiliated Hospital, Anhui University of Chinese Medicine, between May 2018 and December 2018 were enrolled. The inclusion criteria consisted of a WD diagnosis with liver involvement according to the Leipzig standard [18] but no hypersplenism at the time of enrollment. Hypersplenism was defined as a leukocyte count < 3500/µL and/or a platelet count < 7.5 × 10^4^/µL [5]. The exclusion criteria were as followed: the presence of other liver diseases (virus hepatitis, alcoholic liver disease, immune liver disease, et al.); severe liver inflammation indicated by ALT elevation greater than five times upper normal limits [19]; failure in liver 2D-SWE assessment; incomplete clinical data at enrollment or during follow-up; did not undergo regular follow up as required; and lost during follow-up. This prospective study was approved by the Institutional Review Board of Ethics Committee of the First Affiliated Hospital, Anhui University of Chinese Medicine (2018AH-08), and informed consent was obtained from all participants.

### Clinical and imaging data collection at baseline

In the current study, the starting point for each patient was defined as the day when the patient underwent liver 2D-SWE. At baseline, clinical data including sex, age, body mass index (BMI), presence of Kayser-Fleischer (K-F) ring, fibrosis index based on four factors (FIB-4), and aminotransferase/platelet count ratio index (APRI) were collected. Laboratory results obtained within one week of patient enrollment were considered valid. The portal vein diameter and spleen thickness measured by ultrasound, as well as the liver stiffness assessed by 2D-SWE, were documented.

### Assessment of liver stiffness by 2D-SWE

Liver 2D-SWE measurement was performed upon enrollment for each patient. The Mindray-Resona 7 ultrasonic instrument with the SC5-1U probe (Mindray, China) was used and the 2D-SWE mode was adopted. The 2D-SWE measurement was performed by one experienced radiologist (8-year experience of abdominal ultrasound) who had performed more than 300 liver ultrasound examinations in the last two years and at least 50 liver 2D-SWE examinations in the last six months.

**Fig. 1:**
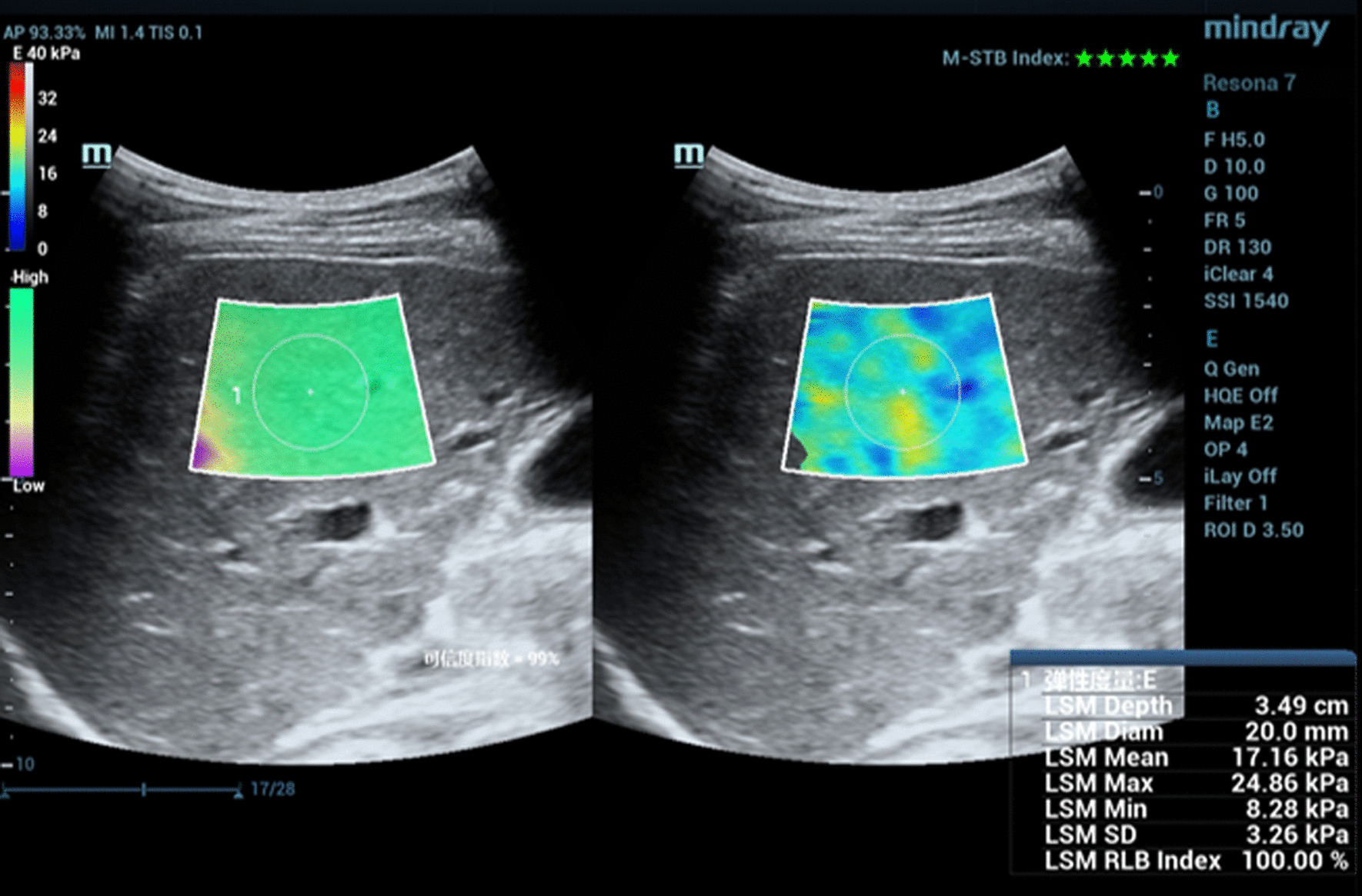
Male WD patient, 26, with an average right liver lobe elasticity of 17.16 kPa

Patients were in a fasting state on the morning of the liver 2D-SWE examination. During the 2D-SWE examination, measurements were performed with the WD patient in the supine (76 patients) or slight lateral decubitus position with 30° (14 patients) [19], with the right hand lifted to fully expose the intercostal space. Liver 2D-SWE images were obtained from the right liver lobe through the right intercostal space. The image depth was controlled at 8–10 cm, and the size of the liver 2D-SWE sampling frame was adjusted to 4.0 × 3.0 cm, placed at 1.0 cm below the liver capsule to keep it away from the liver’s great vessels. During 2D-SWE examination, patients were required to hold their breath for 5–6 s in the natural breathing state. The elasticity value was measured when the motion stability index (M-STB) was of five green asterisks and when the reliability map (RLB) changed from purple to green [19]. During measurements, the circular region of interest (ROI) diameter was adjusted to 2.0 cm. Data are displayed in kPa. The average ROI elasticity obtained from each measurement was considered the recorded value of the current measurement (Fig. 1). The measurements were continuously repeated five times, and the median of the recorded values was considered as the patient’s final liver elasticity value. To obtain more accurate and consistent measurement data, we implemented quality control for images and measurements. If the interquartile range (IQR)/median (Med) was higher than 30% after repeated measurements for five consecutive times, the measurement was considered as failed.

### Treatment and follow-up strategy

All WD patients received de-coppering therapy during the follow-up period. Patients regularly received dimercaptosuccinic acid (DMSA) orally at a dose of 10 mg/kg/d, bid.

After enrollment, patients were routinely followed up every month. At each visit, leukocyte and platelet counts were assessed to determine whether the patient developed hypersplenism [5]. Patients were prospectively followed up for at least 24 months or until they developed hypersplenism.

### Statistical analyses

Measurement data conforming to normal distribution are expressed as means ± SD, and intergroup comparison was performed using a t-test. Measurement data that did not present normal distribution are expressed as medians (IQR), and the Mann–Whitney U test was adopted for inter-group comparison. Enumeration data were expressed as n (%), and inter-group comparison was performed using χ^2^ or Fisher’s exact tests.

Hypersplenism cumulative incidence was determined based on the hazard ratios derived from Kaplan–Meier survival curves. Cox regression analyses were applied to evaluate the hypersplenism risk factors in WD patients. The receiver operating characteristic curves (ROC) and area under the curves (AUC) were used to calculate the efficacy hypersplenism prediction of liver stiffness in WD patients. Additionally, the threshold, as well as its sensitivity and specificity, were determined by the maximum Youden index. Based on the obtained threshold, a Log-rank test was applied to compare and analyze whether significant differences were present when evaluating the hypersplenism risk between the two groups of WD patients divided by the 2D-SWE threshold. SPSS 25.0 was used for all data analyses. Differences were considered statistically significant when *p* < 0.05.

## Results

### General baseline information of the 90 WD patients

**Fig. 2:**
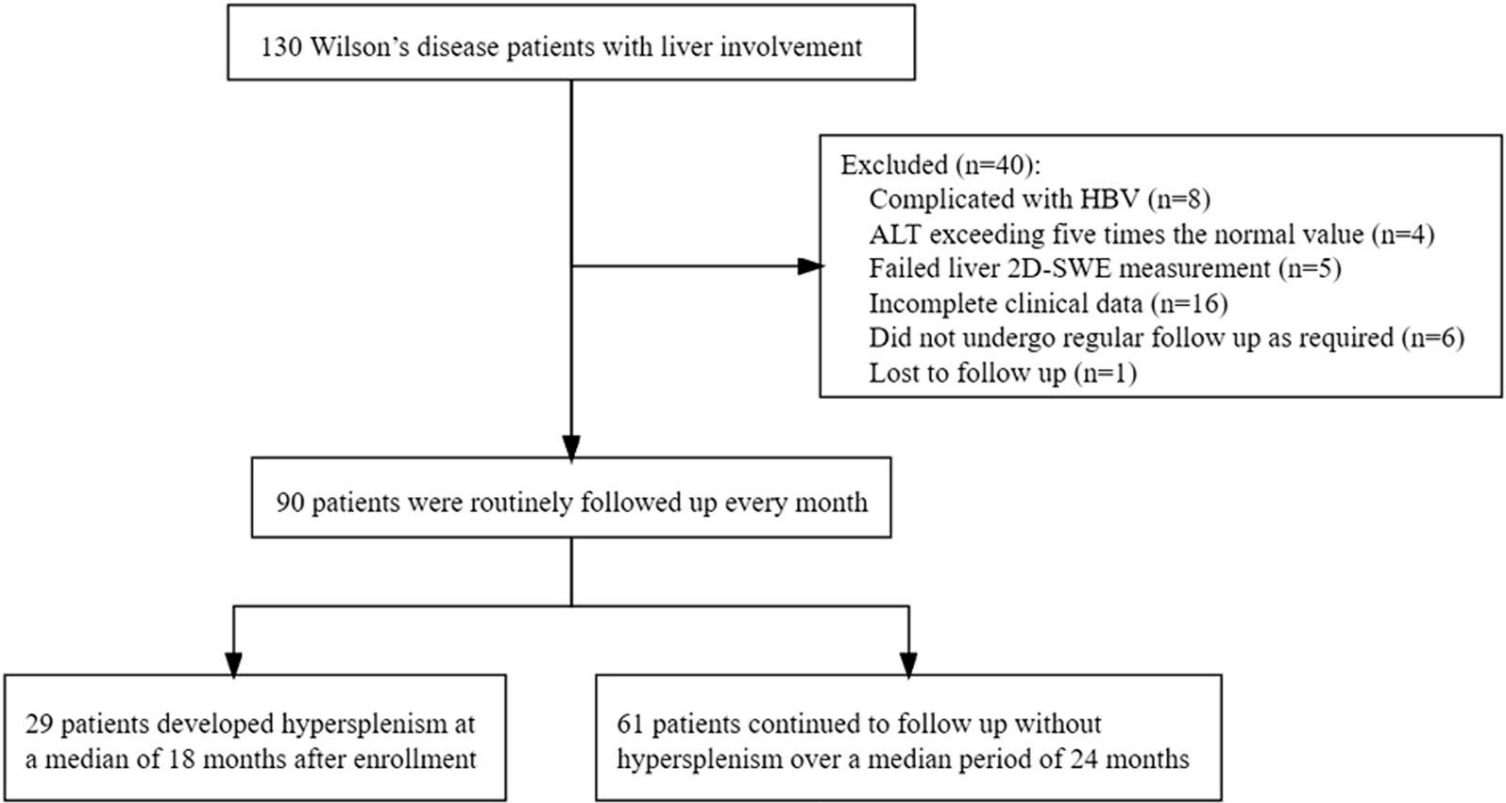
Flowchart of the study

Ninety WD patients were eventually included in this study (Fig. 2). Among them, 49 (54.4%) were males and 41 (45.6%) were females, with an average age of 29.8 ± 10.6 years (range: 14–63 years). General baseline information of the 90 patients is summarized in Table 1. The percentage of liver cirrhosis in the hypersplenism group(n = 29) and non-hypersplenism group(n = 61) were 24.1% (7/29) and 19.7% (12/61) respectively.

**Table 1:** Baseline general information of 90 patients with WD

Factors	The whole cohort	Hypersplenism group	Non-hypersplenism group
Number n (%)	90 (100)	29 (32.2)	61 (67.8)
Male n (%)	49 (54.4)	15 (51.7)	34 (55.7)
Age (years)	29.77 ± 10.62	27.34 ± 7.53	30.92 ± 11.70
BMI (kg/m^2^)	21.46 ± 1.65	21.08 ± 1.78	21.64 ± 1.57
K-F ring	65 (72.2)	20 (69.0)	45 (73.8)
FIB-4	1.47 ± 0.20	1.48 ± 0.20	1.47 ± 0.20
APRI	0.74 ± 0.10	0.73 ± 0.10	0.74 ± 0.10
Diameter of portal vein (mm)	11.67 ± 1.80	12.41 ± 2.03	11.31 ± 1.58
Spleen thickness (mm)	51.88 ± 9.02	53.38 ± 5.14	51.16 ± 10.33
Liver stiffness (kPa)	10.10 ± 2.90	12.38 ± 2.77	9.00 ± 2.28

BMI, body mass index; K-F ring, Kayser-Fleischer ring; FIB-4, fibrosis index based on four factors; APRI, aminotransferase/platelet count ratio index

### Reliable liver stiffer assessment by 2D-SWE in WD patients

Five WD patients failed during the liver 2D-SWE examination. They included two patients that did not hold their breaths in the 2D-SWE process, two that presented involuntary movements since neurologic manifestation, and one with 2D-SWE measurements with IQR/M > 30%. We obtained reliable liver stiffer measurements in 90 (94.7%) of 95 patients.

### Clinical outcome of the follow-up

**Fig. 3:**
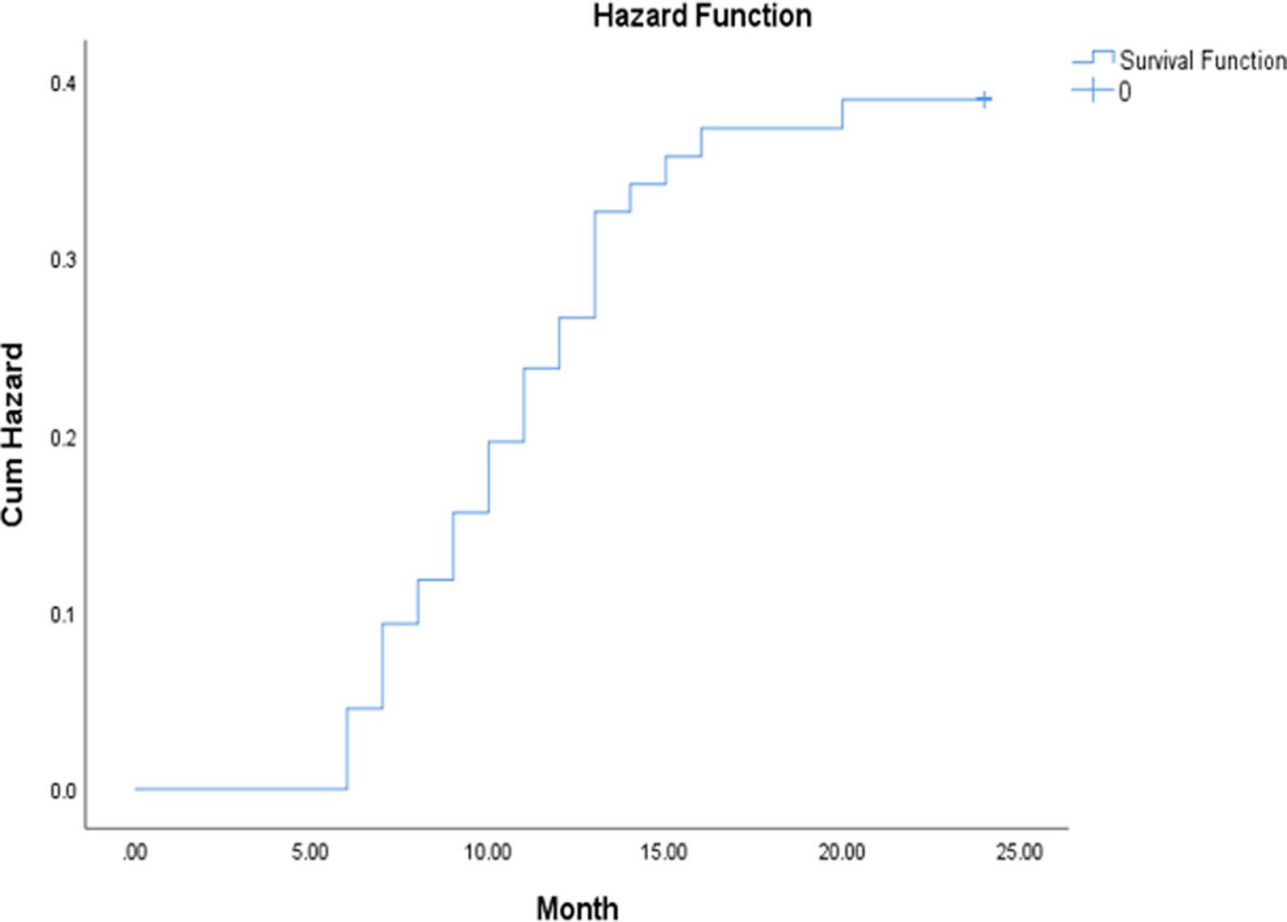
Cumulative hypersplenism incidence in WD patients

During follow-up, 29 (32.2%) patients developed hypersplenism. The median time between the enrollment and the hypersplenism occurrence was 18 months (range, 6–24 months). The cumulative hypersplenism incidence at 6, 12, and 18 months was 4.4%, 21.1%, and 31.1%, respectively (Fig. 3). The remaining 61 (67.8%) patients were continued to follow up without hypersplenism upon a median period of 22 months (range,6–24).

### Risk factors associated with hypersplenism in WD patients

The Cox regression analyses revealed that age, portal vein diameter, and liver stiffness were independent predictive factors associated with hypersplenism (Table 2). The Cox regression value for the liver stiffness was 0.325, which suggested that with every 1.0 kPa increase in the liver stiffness, the hypersplenism risk increase by 32.5%.

**Table 2:** Cox regression analysis of risk factors for hypersplenism in WD patients

Factors	B	*P*	RR value	95% CI	
				Lower limit	Upper limit
Age	− 0.060	0.007	0.942	0.901	0.984
Diameter of the portal vein	0.244	0.012	1.277	1.055	1.545
Liver stiffness	0.325	0.000	1.385	1.231	1.557

RR, relative risk; CI, credibility interval

### Value of the liver stiffness measured by 2D-SWE to predict hypersplenism

**Fig. 4:**
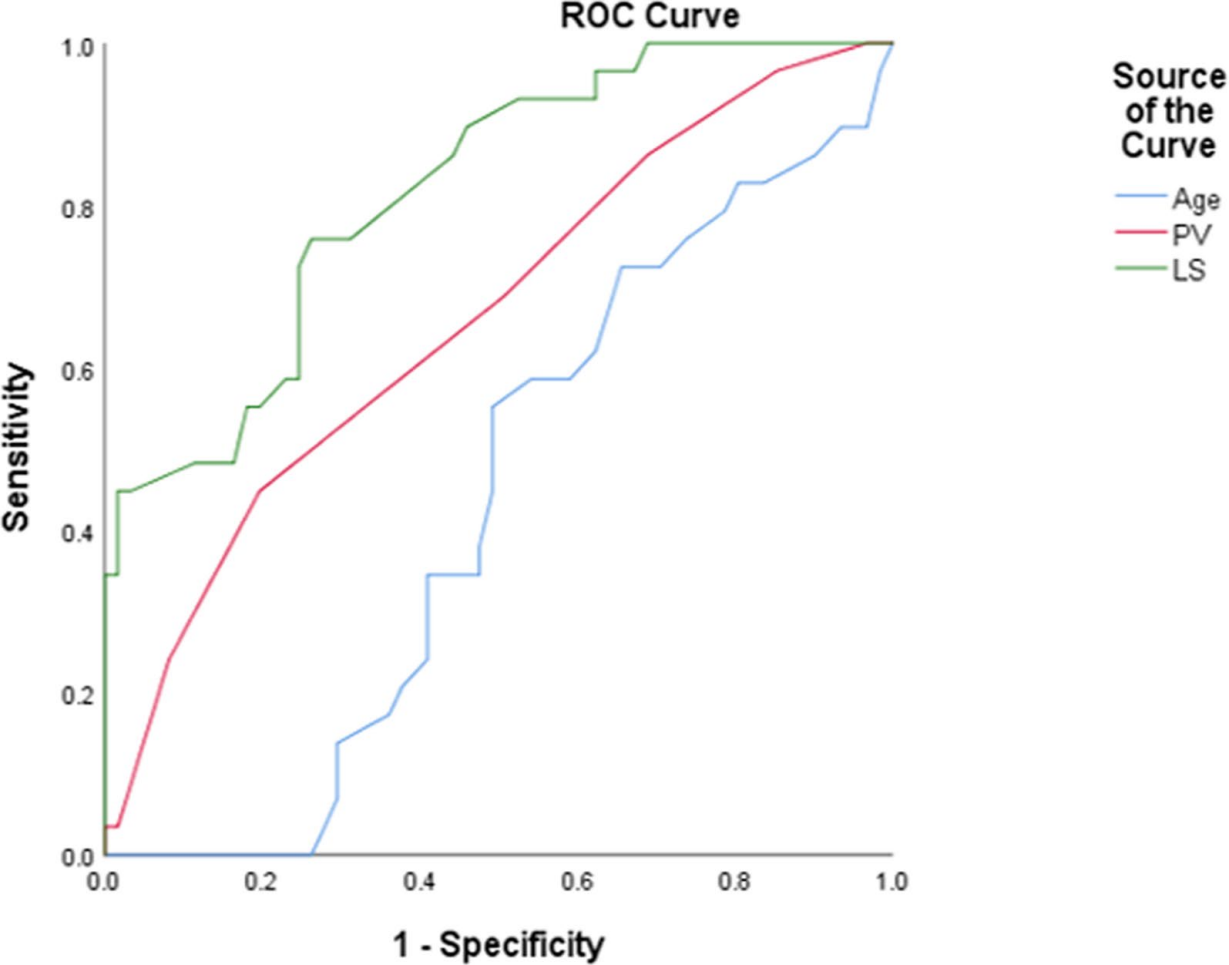
Value of age, portal vein diameter, and liver stiffness measured by 2D-SWE to predict hypersplenism in WD patients

The ROC analyses reported that the AUCs to predict hypersplenism in the WD patients were 0.433 (95% CI 0.314–0.553) for age, 0.662 (95% CI 0.541–0.782) for spleen vein diameter, and 0.817 (95%CI: 0.727–0.908) for liver stiffness (Fig. 4). Thus, liver stiffness had a superior predictive ability compared to portal vein diameter and age (*p* < 0.05). The optional liver stiffness cutoff value to predict hypersplenism in WD patients was 10.45 kPa, with sensitivity and specificity of 75.9% and 73.8%, respectively.

**Fig. 5:**
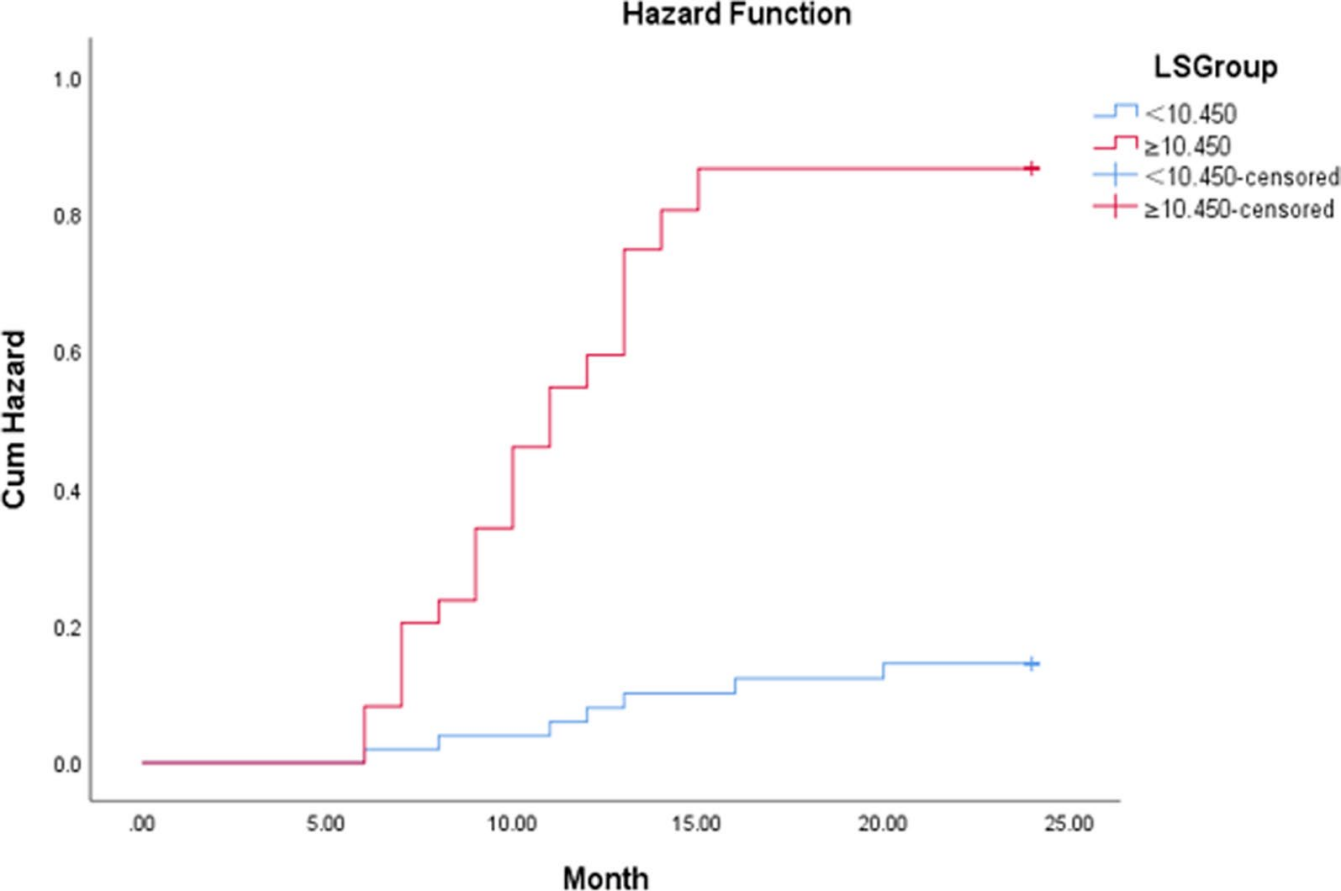
Hypersplenism risk in WD patients in both high- risk (≥ 10.45 kPa) and low-risk (< 10.45 kPa) groups

**Fig. 6:**
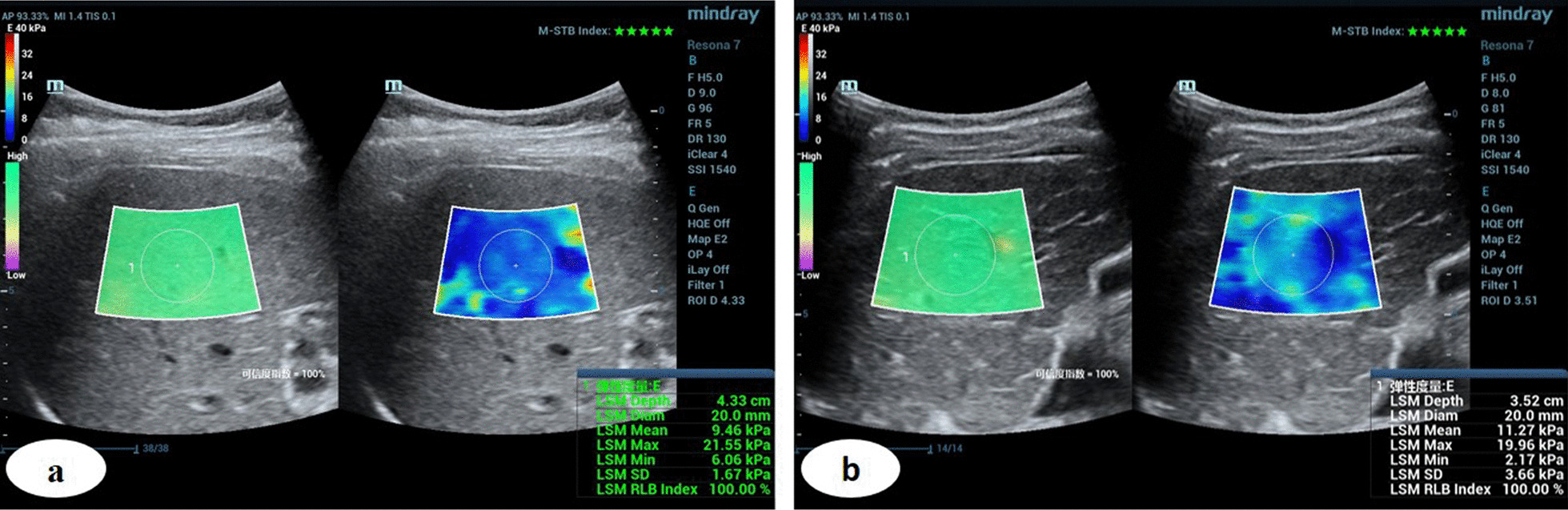
Baseline 2D-SWE measurement of liver stiffer in two WD patients with different risk of hypersplenism. **a** The WD patient's right liver lobe elasticity was 9.46 kPa, followed up for 24 months with no hypersplenism occurrence. **b** The WD patient's right liver lobe elasticity was 11.27 kPa with hypersplenism development at the 13th follow-up month

To determine the possible association between liver stiffness and the time point of hypersplenism development, the 90 WD patients were divided into high-risk (liver stiffness ≥ 10.45 kPa, n = 38) and low-risk (liver stiffness < 10.45 kPa, n = 52) groups, based on the cutoff value of 10.45 kPa. The cumulative hypersplenism incidence in both groups is shown in Fig. 5. The hypersplenism incidence in the high-risk group was significantly higher compared to the low-risk group (57.9% vs. 13.5%, *p* < 0.001). The median time between enrollment and the hypersplenism development for the high-risk group was 15 months (range, 6–24 months), and for the low-risk group was 22 months (range, 6–24 months) (*p* < 0.001) (Fig. 6) (Table 3).

**Table 3:** Incidence of hypersplenism in the subgroups with different liver stiffness

Groups	Liver stiffness measured by 2D-SWE	Incidence of hypersplenism	The median time of hypersplenism occurrence (month)	*χ*^*2*^	*P*
High-risk group	≥ 10.45 kPa	57.9% (22/38)	15	22.296	< 0.001
Low-risk group	< 10.45 kPa	13.5% (7/52)	22		

## Discussion

In the current study, we explored the value of liver stiffness assessed by 2D-SWE to predict hypersplenism in WD patients. We demonstrated that the assessment of liver stiffness using 2D-SWE is effective for the prospective prediction of hypersplenism in WD patients. Furthermore, WD patients with liver stiffness ≥ 10.45 kPa have a significantly higher hypersplenism risk that might develop in a relatively short term. These findings enabled prompt stratification of WD patients with high or low hypersplenism risk.

We found that age, portal vein diameter, and liver stiffness were independent risk factors for hypersplenism in WD patients. Furthermore, the liver stiffness showed a higher AUC value of 0.817 when compared to age and portal vein diameter. In a previous study, 4D flow MRI was used to predict hypersplenism occurrence in 20 patients. Keller et al. found that the AUC value for splenic artery blood flow was 0.792 [20], slightly lower than that of liver stiffness reported in our current study. MR imaging requires contrast agent injection, has a high cost, and excludes patients with incompatible implanted devices. Besides, WD patients typically develop neurological and psychiatric symptoms during disease progression and might not tolerate MRI examination. Therefore, MRI is a nonideal modality for the assessment of WD patients. Our study suggested that the liver stiffness measured by 2D-SWE can be used to assess the hypersplenism risk in WD patients with high effectiveness and feasibility.

WD is characterized by copper metabolic dysfunction, excessive copper deposition in liver tissues leading to hepatocyte fatty degeneration, chronic inflammation, liver fibrosis, and liver cirrhosis [21]. The WD pathological process is consistent with other chronic liver diseases. The 2D-SWE is a two-dimensional ultrasound-based technology to measure tissue stiffness. It enables real-time analysis of liver stiffness and can uncover the underlying pathological processes of liver diseases. Recently, several studies demonstrated that liver stiffness assessed by 2D-SWE was an alternative tool for physicians to predict disease progression, such as CSPH, EV, and GV [14, 15, 17, 22], avoiding hepatic venous pressure gradient (HPVG) and endoscopy. This is of clinal interest since HPVG and endoscopy are invasive procedures varices [23]. In a previous meta-analysis with 746 liver disease patients, it was suggested that 2D-SWE can be used to forecast portal hypertension with 15.2–24.6 kPa cutoffs, with sensitivities ranging from 78 to 90% and specificities varying from 83 to 89% [20]. Few studies have used 2D-SWE to predict hypersplenism so far. Possibly, 45% of liver diseases (e.g., virus hepatitis) patients complicated with portal hypertension manifested EV and GV but just 7.7% developed hypertension [23]. On the contrary, WD patients complicated with portal hypertension manifested hypersplenism with high incidence of 34% [3]. Our results confirmed that 2D-SWE measurement of liver stiffness in WD patients can effectively predict hypersplenism, extending the application of 2D-SWE in predicting liver diseases complications. We found that the cut-off value of liver stiffness was 10.45 kPa to predict hypersplenism in WD patients, with a sensitivity and specificity of 75.9% and 73.8%, respectively [24]. The discrepancy in cutoff values between our study and the previous meta-analysis can be partially explained by the difference in the average age of patients enrolled in the two studies. The average age of the 746 patients in the aforementioned meta-analysis was 53–71, older than those in our current study (29.8 years).

When compared with patients with liver stiffness < 10.45 kPa, those with ≥ 10.45 kPa had an increased hypersplenism risk (RR 1.385). Besides a relatively higher incidence of hypersplenism (57.9% vs. 13.5%), the median time between baseline and the time points of hypersplenism development in the high-risk patients with liver stiffness ≥ 10.45 kPa was significantly shorter (14 months vs. 20 months, p < 0.001). These results implied that high-risk patients might develop hypersplenism in a relatively short term. Portal hypertension and hypersplenism might be reversible in some WD patients when given glutathione (GSH) intravenously at a dose of 1.8 g in the early stage of hypersplenism [25, 26]. Therefore, the high risk WD patients need more frequent follow-up, allowing for physicians to timely diagnose hypersplenism and adjust treatment options [25, 27]. Our study highlighted that the liver stiffness assessed by 2D-SWE can be applied to monitor liver progression of WD patients by stratifying hypersplenism risk.

Our study has several limitations. Due to the rare occurrence of WD, our results are based on only one medical center. Moreover, we used 2D-SWE data collected by a single experienced radiologist, since guidelines and recommendations state that the intra-observer reproducibility of liver 2D-SWE assessment is excellent [28]. Furthermore, we investigated other laboratory indicators but only FIB-4 and APRI were included for analyses. FIB-4 and APRI are commonly used to monitor liver fibrosis in chronic hepatitis B and C patients as well as WD patients 29 [28]. Whether the 2D-SWE prediction efficacy is improved when more laboratory indicators are included in analyses needs further investigation.

## Conclusion

Overall, WD patients with liver stiffness ≥ 10.45 kPa presented a relatively higher risk of hypersplenism. Monitoring liver stiffness with 2D-SWE is essential for WD patients to early diagnose hypersplenism, guide clinical treatment and avoid postoperative complications.

## Data Availability

The datasets supporting the conclusions of this article are available from the corresponding author on reasonable request.

## Abbreviations

2D-SWE: Two-dimensional real-time shear wave elastography
WD: Wilson’s disease
PVT: Portal vein thrombosis
OPSI: Overwhelming post-splenectomy infection
4D: Four-dimensional
MRI: Magnetic resonance imaging
TE: Transient Elastography
CSPH: Clinically significant portal hypertension
HRV: High-risk varices
EV: Esophageal varices
GV: Gastric varices
cACLD: Advanced chronic liver disease
BMI: Body mass index
K-F ring: Kayser-Fleischer ring
FIB-4: Fibrosis index based on four factors
APRI: Aminotransferase/platelet count ratio index
M-STB: Motion stability index
RLB: Reliability map
ROI: Region of interest
IQR: Interquartile range
Med: Median
DMSA: Dimercaptosuccinic acid
ROC: Receiver operating characteristic curve
AUC: Area under the curve
GSH: Glutathione

## References

[1] Poujois A, Woimant F (2018). Wilson's disease: a 2017 update. Clin Res Hepatol Gastroenterol.

[2] Gheorghe L, Popescu I, Iacob S, Gheorghe C, Vaidan R, Constantinescu A (2004). Wilson's Disease: a challenge of diagnosis. The 5-year experience of a tertiary centre. Rom J Gastroenterol.

[3] Roberts EA, Socha P (2017). Wilson disease in children. Handb Clin Neurol.

[4] Zhong HJ, Sun HH, Xue LF, McGowan EM, Chen Y (2019). Differential hepatic features presenting in Wilson disease-associated cirrhosis and hepatitis B-associated cirrhosis. World J Gastroenterol.

[5] Ikegami T, Soejima Y, Taketomi A, Kawanaka H, Yoshizumi T, Shimada M (2009). Hypersplenism after living donor liver transplantation. Hepatogastroenterology.

[6] Li LY, Yang WM, Chen HZ, Wu YH, Fang X, Zhang J (2015). Successful splenectomy for hypersplenism in Wilson's disease: a single center experience from China. PLoS ONE.

[7] Targarona EM (2008). Portal vein thrombosis after laparoscopic splenectomy: the size of the risk. Surg Innov.

[8] Jones P, Leder K, Woolley I, Cameron P, Cheng A, Spelman D (2010). Postsplenectomy infection—strategies for prevention in general practice. Aust Fam Phys.

[9] Li LY, Chen HZ, Bao YC, Yu QS, Yang WM (2018). Successful treatment of hypersplenism in Wilson's disease by partial splenic embolization. J Investig Surg.

[10] Collins JD, Ayache Bou JM, Semaan E, Salem R, Carr JC, Markl M (2015). Quantitative assessment of splenic hemodynamics at 4D flow MRI to diagnose hypersplenism associated thrombocytopenia. J Vasc Interv Radiol.

[11] Shen Y, Zhou C, Zhu G, Shi G, Zhu X, Huang C (2017). Liver stiffness assessed by shear wave elastography predicts postoperative liver failure in patients with hepatocellular carcinoma. J Gastrointest Surg.

[12] Jeong JY, Sohn JH, Sohn W, Park CH, Kim TY, Jun DW (2017). Role of shear wave elastography in evaluating the risk of hepatocellular carcinoma in patients with chronic hepatitis B. Gut Liver.

[13] Yoon HM, Kim SY, Kim KM, Oh SH, Ko GY, Park Y (2017). Liver stiffness measured by shear-wave elastography for evaluating intrahepatic portal hypertension in children. J Pediatr Gastroenterol Nutr.

[14] Procopet B, Berzigotti A, Abraldes JG, Turon F, Hernandez-Gea V, Garcia-Pagan JC (2015). Real-time shear-wave elastography: applicability, reliability and accuracy for clinically significant portal hypertension. J Hepatol.

[15] Grgurevic I, Bokun T, Mustapic S, Trkulja V, Heinzl R, Banic M (2015). Real-time two-dimensional shear wave ultrasound elastography of the liver is a reliable predictor of clinical outcomes and the presence of esophageal varices in patients with compensated liver cirrhosis. Croat Med J.

[16] de Franchis R, Baveno VIF (2015). Expanding consensus in portal hypertension: report of the Baveno VI Consensus Workshop: stratifying risk and individualizing care for portal hypertension. J Hepatol.

[17] Fofiu R, Bende F, Popescu A, Sirli R, Miutescu B, Sporea I (2021). Assessing Baveno VI criteria using liver stiffness measured with a 2D-shear wave elastography technique. Diagnostics (Basel).

[18] Ferenci P, Caca K, Loudianos G, Mieli-Vergani G, Tanner S, Sternlieb I (2003). Diagnosis and phenotypic classification of Wilson disease. Liver Int.

[19] Barr RG, Wilson SR, Rubens D, Garcia-Tsao G, Ferraioli G (2020). Update to the society of radiologists in ultrasound liver elastography consensus statement. Radiology.

[20] Keller EJ, Kulik L, Stankovic Z, Lewandowski RJ, Salem R, Carr JC (2017). JOURNAL CLUB: four-dimensional flow MRI-based splenic flow index for predicting cirrhosis-associated hypersplenism. AJR Am J Roentgenol.

[21] Ferenci P (2004). Pathophysiology and clinical features of Wilson disease. Metab Brain Dis.

[22] Maruyama H, Kobayashi K, Kiyono S, Sekimoto T, Kanda T, Yokosuka O (2016). Two-dimensional shear wave elastography with propagation-based reliability assessment for grading hepatic fibrosis and portal hypertension. J Hepatobiliary Pancreat Sci.

[23] Fofiu R, Bende F, Popescu A, Sirli R, Lupusoru R, Ghiuchici AM (2021). Spleen and liver stiffness for predicting high-risk varices in patients with compensated liver cirrhosis. Ultrasound Med Biol.

[24] Suh CH, Kim KW, Park SH, Lee SS, Kim HS, Tirumani SH (2018). Shear wave elastography as a quantitative biomarker of clinically significant portal hypertension: a systematic review and meta-analysis. Am J Roentgenol.

[25] Aggarwal A, Bhatt M (2018). Advances in treatment of Wilson disease. Tremor Other Hyperkinet Mov (N Y).

[26] Dietrich CF, Bamber J, Berzigotti A, Bota S, Cantisani V, Castera L (2017). EFSUMB guidelines and recommendations on the clinical use of liver ultrasound elastography, update 2017 (long version). Ultraschall Med.

[27] Kozic DB, Semnic R, Petrovic I, Svetel M, Ostojic J, Kostic VS (2012). Are irreversible morphological [corrected] signs of portal hypertension in neurological form of Wilson's disease associated with treatment delay? A pilot study. Acta Neurol Belg.

[28] Hwang J, Yoon HM, Jung AY, Lee JS, Kim KM, Oh SH (2020). Diagnostic performance of ultrasound elastography and serologic fibrosis indices for evaluation of hepatic involvement in Wilson disease. J Ultrasound Med.

